# School-based victimization of transgender youth: A qualitative study

**DOI:** 10.23938/ASSN.1108

**Published:** 2025-09-16

**Authors:** David Martin-Castillo, José Joaquín García-Arenas, María Sánchez-Muñoz, José Antonio Jiménez-Barbero, María del Mar Pastor-Bravo

**Affiliations:** 1 University of Murcia Faculty of Nursing Murcia Spain; 2 Instituto Murciano de Investigación Biosanitaria (IMIB) Murcia Spain; 3 Saint Anthony Catholic University Occupational Therapy Department Murcia Spain; 4 Murcian Health Service Cartagena Mental Health Center Cartagena Murcia Spain

**Keywords:** Transgender persons, Students, Mothers, Bullying, Qualitative Research, Personas transgénero, Estudiantes, Madres, Acoso Escolar, Investigación Cualitativa

## Abstract

**Background:**

Evidence indicates that transgender adolescents are at heightened risk of experiencing violence during their school years. This study aims to explore victimization experiences from the perspective of transgender students and their mothers.

**Methods:**

A qualitative phenomenological approach was used, employing in-depth interviews to examine experiences of school victimization among transgender youth and their mothers. Participants were recruited until data saturation was reached (n=10). Interviews were audio-recorded, transcribed, and analysed using thematic content analysis in parallel with ongoing data collection.

**Results:**

Transgender children and adolescents, along with their mothers, described various victimization forms in school settings, including physical and verbal abuse, and cyberbullying. These experiences were associated with significant biopsychosocial consequences, such social withdrawal, violent behaviour, depression, and suicidal ideation.

**Conclusions:**

The findings in this study highlight the urgent need to understand and address the diverse forms of victimization faced by transgender students. The study underscores the importance of implementing comprehensive anti-bullying strategies, including awareness campaigns, peer support systems, targeted protocols, and specialized training for school personnel.

## RESUMEN

**Fundamento:**

La evidencia científica ha demostrado que las personas transgénero corren el riesgo de ser víctimas de violencia durante su etapa escolar. El presente estudio busca explorar las experiencias de victimización del alumnado transgénero desde su propia perspectiva y la de sus familias.

**Métodos:**

Estudio fenomenológico cualitativo mediante entrevistas en profundidad para explorar las experiencias de victimización del alumnado transgénero y sus madres. Se reclutó a los participantes hasta alcanzar la saturación de datos (n=10). Las narrativas se grabaron y transcribieron, aplicando el análisis de contenido temático en paralelo a la recopilación de más entrevistas.

**Resultados:**

Niñas, niños y adolescentes transgénero y sus madres describieron diferentes experiencias de victimización sufridas en la escuela: física, verbal y acoso en redes sociales. Estas experiencias se asociaron con consecuencias para su salud a nivel biopsicosocial, como el aislamiento social, las conductas violentas, los intentos autolíticos y la depresión.

**Conclusiones:**

Los resultados de este estudio sugieren la importancia de analizar las diferentes experiencias de victimización que sufre el alumnado transgénero en la escuela. Es necesario desarrollar estrategias que contribuyan a abordar el acoso escolar en personas trans, como la sensibilización, la tutoría entre iguales, el protocolo contra el acoso escolar, la sensibilización y la formación.

## INTRODUCTION

Transgender people are individuals whose gender identity does not align with the sex assigned to them at birth. This incongruence between their experienced gender and assigned sex can profoundly impact the person’s life at various stages^1^. Specifically, those assigned male at birth who identify and live as female are referred to as trans women. Another category, often termed *queer*, encompasses individuals whose gender identity exists outside the traditional binary of *male* and *female*. While *queer* may broadly include members of the LGBT community, it particularly denotes non-normative identities that challenge conventional gender and sexual norms. In contrast, cisgender refers to individuals whose gender identity corresponds with their assigned sex at birth^2^.

The gender transition process for transgender individuals involves multiple stages. Physical transition refers to aligning one´s physical appearance with their gender identity, while *social transition* encompasses communicating one´s identity to family and friends, changing one´s name, and asserting the use of appropriate pronouns^3^. These transitions often begin in childhood or adolescence and can profoundly affect one´s psychosocial context^4^.

In Spain, recent data indicate the prevalence of transgender is approximately 1 in 1,000^5^, consistent with estimates from countries such as the United States, Australia, Malaysia, New Zealand, and the United Kingdom^5^^-^^8^. Moreover, the transition process presents significant challenges to hospital-level healthcare delivery^9^.

Transgender children frequently encounter school-based violence, defined as repeated physical and/or psychological aggression directed by peers at targeted students. This hostile behaviour traps victims in a cycle that is difficult to escape from without external intervention^2^. Such experiences can cause diminished self-esteem, heightened anxiety, and symptoms of depression, all of which hinder academic integration and learning development^10^^-^^12^. Worryingly, research consistently shows that transgender youth face higher rates of bullying compared to their cisgender peers^13^^-^^15^.

In our study, we adopt the concept of stigma-based bullying, acknowledging that various stigma-related factors - such as social dominance orientation, stereotypes, and prejudice - partially contribute to its occurrence^16^.

Therefore, to effectively combat stigma-based bullying, it may be necessary to address the underlying contributing factors. Our theoretical framework was adapted from Urie Bronfenbrenner’s (1986) ecological model, which emphasizes an environmental approach to individual development, considering the multiple contexts that influence cognitive, moral, and relational growth^17^.

This model situates the individual who engages in stigma-based bullying within a broader network of societal layers, institutional structures, and interpersonal relationships, highlighting how influences at each level can contribute to such behaviour^18^. Finally, stigma-based bullying manifests in diverse forms and has a significant impact on various domains of health and well-being in victimized youth, including their academic performance^19^.

School victimization has significant negative consequences for transgender individuals^20^. Transgender adolescents are twice as likely to experience school absenteeism due to the impact of such victimization^3^. Moreover, it hinders their educational progress and limits their future professional opportunities^21^.

Scientific evidence identifies a range of factors that influence the victimization of transgender students in school settings, including the level of support received from family, peers, teachers, and the broader school institution^15^^,^^22^. Several authors emphasize the need for measures that promote the safety and well-being of transgender students, and prevention programs aimed at addressing prejudice-driven bullying targeting transgender youth^20^^,^^23^. In particular, the literature underscores the importance of implementing school-based prevention programs against bullying based on prejudice towards transgender people^10^. Similarly, there is a recognized need to develop targeted initiatives to help prevent school victimization among transgender and gender-diverse children and adolescents^20^.

Although there is substantial research on bullying within this population, few studies have examined the phenomenon through qualitative lens. One notable study conducted in England between 2015 to 2016 explored gender diversity among youth aged 12 to 14 and investigated the role of schools as environments for learning about and supporting sexual and gender diversity^23^. In a related effort, the Austin Independent School District in England published a report comparing the victimization experiences of transgender students to those of their cisgender peers^20^.

Given the gap in knowledge identified in the literature review, we consider it essential to examine the school victimization experiences of transgender individuals and to assess the impact of school-based bullying prevention programs. To the best of our knowledge, the present study is the first conducted in Spain that addresses this issue by incorporating the perspectives of both transgender students and their families.

Before developing the interview instrument, the following research objectives were defined. The primary aim of this study was to explore the school experience of transgender individuals from the viewpoints of trans students and their parents. The secondary objectives were to: (a) describe the forms and frequency of victimization experienced by trans students in school settings; (b) identify the consequences of such victimization; (c) investigate strategies currently used to prevent bullying of transgender children and adolescents in schools; and (d) determine potential areas for intervention, particularly from the perspective of school nursing.

## METHODOLOGY

### Study design and sampling

This study employed a qualitative phenomenological design, selected to gain a deep understanding of the school victimization experiences of transgender individuals through the narratives of the individuals themselves and their families^24^.

The sample consisted of 10 trans students aged 11 to 16 and their families, all residing in the Region of Murcia and the Valencian Community, located in southeastern Spain.

Inclusion criteria for the minors were as follows: self-identification as transgender, being under 18 years of age, current enrolment in a primary or secondary school within the region, and being at least 10 years old to ensure adequate comprehension during the interview. Additionally, both the minor and at least one parent or legal guardian were required to provide consent for participation. Inclusion criteria for family members included being the parent or legal guardian of a transgender minor aged between 10 and 16 years. Minors whose parent(s) or legal guardian(s) declined to participate were excluded from the study.

Purposeful sampling was used. Families were contacted through regional associations supporting transgender individuals and their families. Two families affiliated with these associations were first recruited based on researcher convenience. Subsequently, a snowball sampling technique was employed to identify additional participants until data saturation was achieved. Notably, after the seventh interview, no new codes emerged; in accordance with methodological guidelines^25^, three additional interviews were conducted to confirm saturation. Potential participants were contacted by telephone to verify eligibility, explain the objectives of the study, and, if interested, schedule the interview at a time and location of their choosing.

Although both parents were invited to participate, in all cases only mothers agreed to take part. In one case, both parents declined participation, and their child was therefore excluded from the study.

### Tools and procedure for data collection

Data were collected through open-ended interviews with self-identified transgender children and adolescents and their mothers and conducted between January and July 2020. Based on the study objectives and a prior review of the literature, interview guides were developed - one for the parents and another for transgender children and adolescents. These guides focused on school experience, victimization, and coping strategies.

To ensure clarity and the use of inclusive language, some items in the original guides were revised in collaboration with a member of a transgender family association. The interviews incorporated prompts to facilitate dialogue and encourage elaboration when needed. While the guides provided a structure, the interviews remained flexible; the number and order of questions varied depending on each participant´s experiences and preferences in sharing their story.

Each interview began with a socio-demographic questionnaire to gather relevant background information. The questionnaire included variables such as age, sex assigned at birth, gender identity, race/ethnicity, family structure, school year, average academic performance in the last term, and the occupation and education level of the parents.

The final interviews guides (for both children and parents) covered information the following topics: gender identity, the process of gender transition, the child´s situation and emotional well-being at school, experiences and consequences of school victimization, and the resources and strategies implemented in schools to address bullying of transgender students.

Prior to initiating the interviews, the study objectives and methodology were explained to each participant. This information was conveyed orally and in writing through a pre-interview discussion and a study information sheet. Voluntary participation was emphasized as a prerequisite for involvement, and explicit permission was requested to audio record the interviews. Subsequently, participants were provided with a copy of the *Informed Consent Statement* for parents and the *Informed Assent Statement* for minors. These documents were duly completed and signed. Participants were assured that their confidentiality and anonymity would be maintained at all times. To safeguard confidentiality, each interview was coded using and identifier; the testimonies of each interviewee with children or adolescents were labelled with the letter “C”, and those with mothers with the letter “M”, followed by a number indicating the sequence of the interview, within the same family unit. The study received ethical approval from the Ethics Committee of the University of Murcia (ID: 2610/2019).

To minimize bias, interviews were conducted with children before their mother, ensuring that children did not feel their narratives had already been shared. The interviewer began each session by introducing himself and sharing his personal motivation for the study - specifically, the lack of research on bullying experiences among transgender youth, which had inspired his doctoral thesis. The interviewer also encouraged participants to ask questions, respond voluntarily, and withdraw from the interview at any time without consequences.

Semi-structured interviews were used to explore the participants´ school experiences. The questions were nor presented in fixed order or in identical wording across interviews. In alignment with grounded theory methodology, data collection and analysis were conducted concurrently, allowing interview questions and strategies to evolve in response to emerging categories and concepts.

All interviews were conducted individually and in person in a quiet chosen by the participants - most commonly at their homes (n=9), and in one case in a public library (n=1). Due to COVID-19 restrictions, three interviews were conducted via video conferencing. No financial compensation was provided for participation. Interview duration ranged from 30 to 50 minutes for children and from 45 to 90 minutes for mothers. All interviews were audio-recorded with participants´ consent using a digital recorder. In addition, field notes capturing participants´ non-verbal communication and key points of discussion were documented in a field diary. Following the interviews, recordings were transcribed verbatim and integrated with the corresponding field diary notes.

Given that interviews were co-constructed between interviewer and interviewer^32^, the positionality of the interviewer was acknowledged as a key component for understanding the narrative generated. All interviews and participant recruitment were conducted by the first author, who maintained a reflective journal throughout the research to consider his own positionality as a white, young, cisgender male, a nurse, and a doctoral student in health sciences. His familiarity with the topic was informed by prior work on a systematic review on bullying in transgender people. The research team comprised professionals from diverse health disciplines, and with substantial scientific experience and a demonstrated sensitivity to transgender issues, either through academic inquiry or through personal experience.

Furthermore, throughout data collection and analysis, several experts in the field were consulted to critically assess issues of positionality and reflexivity, ensuring that findings were firmly grounded in the data.

### Data analysis

Data analysis was conducted in parallel with ongoing data collection, following the principles of emergent qualitative research design. All identifying information was removed from the transcripts^26^. The analysis was carried out using thematic content analysis. Two authors (DMC and MPB) independently coded the data and reached consensus through discussion on any discrepancies. ATLAS.ti version 8.4 was used to organise and manage the qualitative data throughout the analysis process.

Thematic analysis of data was conducted inductively in three main phases: (i) initial pre-coding and conceptual indexing. The first set of interviews was pre-coded, and an index of preliminary concepts was established to guide subsequent analysis. The researchers read each transcript multiple times to familiarize with the data, taking notes to identify emerging ideas potential and codes; (ii) coding and categorization: all codes were grouped into broader categories. Each researcher independently carried out line-by-line coding for each transcript. The authors subsequently reviewed the transcripts together, discussing the relevance of each code in capturing sensitizing concepts and ensuring the codes authentically reflected participants´ narratives. A content analysis followed, aimed at determining the prevalence of different themes and subthemes^25^; (iii) interpretation and thematic development. Finally, a list of categories and sub-categories was developed, interpreted, and defined^27^. Relationships between categories were explored to establish thematic connections. This process culminated in the 1) *Experiences of school victimization*, 2) *Consequences of school victimization*, and 3) *Coping strategies for bullying in schools*.

## RESULTS

### Participants’ profile

The study included seven transgender boys and three transgender girls, aged between 11 and 16 years, residing in the Region of Murcia and the Valencian Community (Spain), along with their mothers.

All participants self-identified as white. Most live with their parents and siblings. Their education levels ranged from 5^th^ grade of primary school (equivalent to 6^th^ grade in the USA) to the 1^st^ year of baccalaureate (11^th^ grade USA), with the majority enrolled in secondary education by the time this paper was written. Academic performance in the last grading period varied: one participant received a *pass* (C+), two received *sufficient* (C), five received *good* (B), and two achieved and *outstanding* grade (A).

In terms of maternal employment, most were employed, while one was a homemaker and another was unemployed. Regarding educational background, three mothers completed primary education, two secondary education, two vocational training, and two university degrees. Table 1 presents the main socio-demographic characteristics of the participants.

**Table 1:** Socio-demographic characteristics of the participants

Variable	n
*Age (years)*	
11	2
12	1
14	1
15	3
16	3
*Felt gender identity*	
Boy / Male	7
Girl / Female	3
*School level*	
5º *Primaria* (Grade 5)	1
6º *Primaria* (Grade 6)	1
1º *ESO* (Grade 7)	1
2º *ESO* (Grade 8)	1
3º *ESO* (Grade 9)	3
4º *ESO* (Grade 10)	2
1º *Bachiller* (Grade 11)	1
*Marital status of the mother*	
Married	7
Divorced	2
Single	1
*Mothers’ educational level*	
Primary education	3
Secondary education	2
High education	1
Vocational training	2
University education	2

ESO: *Educación Secundaria Obligatoria* (Secondary Education).

### Experiences of victimization at school

Participants described a range of victimization experiences within the school setting. They detailed the locations, individuals involved (particularly classmates), and specific incidents, as well as the responses from parents and school staff. They also shared their emotional reactions to these experiences.

*Types of school bullying*

The majority of participants reported being subjected to various forms of bullying, including verbal harassment, cyberbullying, and occasional physical aggression. Additionally, participants recounted institutional victimization, experiencing discriminatory or harmful behaviour not only from peers but also from members of the school community, sometimes reinforced by the attitudes of other students´ parents. Table 2 presents the testimonies that illustrate these experiences.

**Table 2:** Types of school bullying

Code	Testimonies
*Physical harassment*	*Yes, I was more afraid than sad, I don’t know. I was very scared to go, because sometimes he threatened to hit me (C4).*
	*Well,...a Moroccan boy, he spent two years bothering, insulting, assaulting...even, luckily there was no suspension for my son, but I had to go to school several times, and several times the other person was expelled...but even with abuse (M6).*
	*They pushed him and insulted him [...] No, it was like pushing him and telling him.... You’ll never be a boy because you don’t have what boys have and so on... (M7).*
*Verbal harassment*	*(M6).Well, they picked on him because he had long hair. They even called him ‘you faggot’. Insults (M6).*
	*At school they used to say to me, what are you doing dressing up as a girl, disgusting! A man dressed up as a woman (C9).*
	*He called me as he used to do, he used to call me sick, because of the way I was, he used to pick on me in all the classes, even if I didn’t say anything, any insult was on me (C4).*
	*I remember I was on a school trip and we were on the bus, and another girl told me that a girl was making fun of me because I was dressed like a boy because I wanted to feel cool, and I felt pretty bad, I had a really bad time on this trip (C5).*
	*One day we were in a gym class and we had to go to wash up, and when I went in, there was a girl who was messing with me, and she said to me, what are you doing, coming in here to the girls’ bathroom, she pushed me and slammed the door on my face (C10).*
*Bullying on social media*	*Well, telling me on Instagram that I was a faggot, I was a tranny, and suchlike (C4).*
	*Some time ago I was on WhatsApp and they put me in a group where we were transsexual, bisexual people or things like that and well...there was one who was, I don’t remember what he was, but he loved to bother transsexual people and he started messing with my body (C6).*
	*(C10).There was an application in which you could write and it was anonymous and that person wrote to you and you received the message, but you didn’t know who it was, and sometimes they would say to me ‘gorilla, fat, but what are you doing thinking you’re a boy’, and suchlike (C10).*
	*On social networks I suppose she told you about it, an application, I told her to remove that application and her sister also told her, because they were messing with her in a brutal way, and as you couldn’t know who they was because it was anonymous, so we removed that application from her phone, but it’s the same people who go with her, the only thing is that it was anonymous, you didn’t know who they was (M10).*

*Attitude of classmates’ parents*

In some cases, classmates´ bullying behaviours and prejudices were reinforced or justified by their parents. For example, participant M10 described how a mother excused her son’s bullying based on religion beliefs, stating that her Evangelical faith does not accept transgender people.

*The teacher arranged a meeting with the mother of the other girl and me, the mother did not attend saying she belonged a different religion and that, in her religion, my daughter’s case was not well regarded. So, the teacher gave talks; she said Today we are going to talk about what happened to your classmate. In my class you are all equal. She did things like that (M10).*

*Attitude of teachers*

Most participants report strong support from teachers, who played a positive role in helping transgender children adapt and thrive in the school environment*.*

*The support of all teachers, in general, was quite good. But there was one, who went overboard, she is the one who helped me to change my name on the school lists and everything, and the truth is that I owe her almost everything (C2).*

*They are very willing, the teachers are involved from the very first moment, and I went to talk to her. From the first minute that everything came up, the issue of transition. I spoke to the tutor and the headmistress, and we talked about the whole issue and so on, and they were very willing, they immediately changed the name on the lists (M8).*

However, some children and adolescents also reported instances in which some members of the educational community placed them in embarrassing or uncomfortable situations, making it difficult for them to express their gender identity and hindering their overall development within the school environment.

*Last year, with my little girl, because she liked to wear her trousers, the kind you wear down, and they told her that she had to feminine, they pulled up her trousers in class and then one day I came in at break time and told him/her what was the matter with him/her and that my little girl could dress the way she wanted and that she wasn’t feminine because she didn’t want to (M10).*

Consequences of school victimization

Both students and their mothers described a variety of adverse consequences of school-based victimization on academic performance, emotional well-being, and personal development.

*Psychosocial consequences*

Participants recounted a range of negative psychological and social effects resulting from bullying. These included depressive symptoms, social withdrawal, and in some cases, self-harming behaviours or suicide attempts. Some mothers also observed aggressive behaviour in their children, which they attributed to prolonged victimization. Testimonies are provided in Table 3.

**Table 3:** Testimonies about psychosocial consequences of school victimization

Code	Testimonies
Social isolation/ Seclusion	Well...I don’t like boys in general, nor girls as they are...I simply don’t talk much with my classmates and I spend my classes without saying anything until break time is over and I’m with my partner, so...I have no problem (C6).
	Yes, now they opens up a lot more than before. Some time ago he didn’t use to tell me anything. He didn’t have many friends either, so he didn’t have much to tell, or things that had happened at school, or anything (M6).
Violent behaviour	*She used to hit when they picked on her, because that was an application that she downloaded and there she could have all her class, and others, acquaintances and friends, they told her on it what they didn’t dare to say to her face as she could hit them, because before that was the way she defended herself, hitting, the point was she became a though person, and that’s why now nobody picks on her, because her way is, if you say this to me I’ll hit you (M10).*
	*And I got angry very quickly and the first thing I used to do was punching*
	*the wall, that’s what I used to do (C4).*
	*There was even once, when she apparently got very angry... there was an injury to a teacher, who tried to separate them (M4).*
Depression	*I remember once... I was going out with a guy, just before I realised I was trans, and I told him about, and well, he left me because... he supposedly liked girls, and I was kind of depressed, I thought people didn’t like me if I wasn’t... as I should be, like a girl (C5).*
	*Two years ago, when I said I wanted to be... trans, that I was trans, at that time I was diagnosed with depression, and that came together with the change of gender. And then... it kind of lowered my mood a lot (C10).*
Autolytic attempts	*Apart from that, she has tried twice to commit suicide, taking pills. And then I was very worried, we had to keep medication stored... Well, what happened is that she took many pills twice, in the morning before going to school. Then, of course, the effect occurred there, she started vomiting, she fainted, they had to call an ambulance, and then they called me and the second time exactly the same thing because she had argued with the boy she liked and then, of course, they keep an eye on her (M5).*
	*He took a blister pack of ibuprofen, it was an autolytic attempt, it was related to when they picked on him outside school (M7).*

### Academic consequences

Participants emphasized that their experiences of school victimization had notable impact on their academic performance, which led to declining grades, increased absenteeism, and in some cases, the consideration or decision to change schools as a way to escape abuse (Table 4).

As a result of the bullying and the impact of the violence suffered by these children, some parents or the child consider or decide to change school as a strategy to escape violence.

*Well, I was worried about bullying, we had even to change school, and then to adapt to the new school (M5).*

*This last year she was eager to finish the school year, she would have even changed school (M9).*

### Strategies for addressing bullying in schools

Participants described existing efforts within the schools to address bullying of transgender students (Table 5). However, they also highlighted a lack of specific protocols targeting bullying related to gender identity. Participants emphasized the importance of developing and implementing targeted strategies for transgender children and adolescents. Notable suggestions included: assigning a designated tutor or mentor as a trusted point of contact; pairing students with an older peer as a tutor or support figure; ensuring access to counsellors, tutors, or trained staff members; training and raising awareness among both educational staff and students to reduce stigma and foster an inclusive environment (Table 5).

In some cases, school actions were only initiated after parental demands.

*It had to be when the rest of us said ‘look, so far and no further. So I don’t think they are prepared, they act when the parents are already burnt out and say ‘no no no’. It’s like I don’t want any problems. I don’t know how to explain, the perception I have both at primary school and at high school because normally the headmasters and headmistresses are of a certain age. And I get this feeling (M2).*

*No, we haven’t done anything (C8).*

**Table 4:** Testimonies and outcomes of school victimization

Code	Incident
Avoid attending school/school absenteeism	*I tried not going to school many times because a boy picked on me, insulted me and the others laughed at me. I mean, it’s like, he always had to pick on me in class and I got to a point when I got home and the next day I would tell my mother I don’t want to go to school because I don’t..., I used to give her the same excuse my head hurts my stomach hurts because I didn’t feel like going because of this. I didn’t go to school for more than a week, almost two (C4).*
	*I didn’t feel like going to school because of the situation I was going through (M7).*
	*They picked on her. Once they didn’t let her go to the toilet, in fact, the teacher called me several times because the child didn’t perform well, maybe in a month she didn’t want to go for four days because she cried, they picked on her... Maybe she tried to trick me by saying, my tummy hurts, so she didn’t go to school and then she was normal all morning, and we also have a parents’ application in which I talk directly to the teacher and I would tell the teacher, today she is not very convinced at school, if you saw anything, and then the teacher told me, your daughter is great, she didn’t want to come because of the situation (M10).*
Academic performance	*I felt uncomfortable and that’s why I didn’t want to go to school. I didn’t study at all. My grades went from good to sufficient and fail...then there was a change and the teachers had to talk to my mother (C5).*

**Table 5:** Existing and proposed strategies to prevent and address bullying of transgender children and adolescents

Codes	Testimonies
Existing strategies	
Raising awareness/Training	*They are trained and both teachers and pupils participate in training activities, and specific training on trans issues, a team from Valencia, this is specifically to the Valencian community, so a team from Valencia comes to give sexuality education to the children and they also talk about the trans issue, so there are also materials and things in the library and at the beginning, when my son started the transition, they were doing, working on cards and things with the children, because as I see it a way to avoid bullying or harassment is through the education of children (M1).*
	*Well, in sixth grade we studied gender identities in natural sciences (C2).*
	*The teachers attended a talk about gender identity, because she has told me about it several times, her teacher, but some of them have also attended, as far as I remember one, about gender identity, a lot of young people from a trans association came to give a talk about how they felt and all that (M3).*
Peer tutoring	*Now they have a tutor at school, but they have this for everything, not just for trans children; in other words, a child from a higher grade is a tutor for a child from lower grades and let’s say that they watches over him/her so that there is no bullying; and then they also work a lot on bullying, they are always working on the issue of bullying, doing activities and things, not specifically trans, but with the issue of bullying in general (M1).*
Protocol	*They have a kind of protocol that consists of keeping an eye on the person, being aware where they are, informing their tutor, informing the teaching staff and, above all, the social worker who also moves things around, his high school is very active (M3).*
Proposed strategies	
Awareness raising/Training	*Well, more than talks, some workshops. Real training, not gathering a bunch of people in an assembly hall and telling them that bullying is wrong, because it’s the same old thing. I would make it more like a workshop and make trans people tell their experience, with bullying and so on, where people can have a bit of empathy when it comes to insulting someone, that they have a bit of empathy and think twice (M3).*
	*I think that the talks should be given earlier, to those in 1ºESO (Grade 6) so that they could start to understand the issue, not to those in 4º ESO or Baccalaureate ( Grade 10-11) (M4).*
	*Well, I don’t know, maybe there would be some kind of talk or help, that help should be offered (C6).*
	*Non-binary people should also be mentioned even if they don’t make a lot of noise, at least they start to be heard (M2).*
	*Give more talks, make people aware of the toilets, the changing rooms in PE, to change their shirts and all that (M8).*
	*I think such talks are necessary for people who are closed-minded about these issues (C4).*
	*Well, ... it depends on the teacher in question, but, in general, well, I think that trained, trained, well, maybe they are open-minded, but I don’t think they are really trained (M5).*
	*Parents are the ones who have to instil in their children that they have to respect everything and everyone. At school, there is little that can be done if at home you listen look at this faggot..., look, she is a lesbian. I think it has to be at home. Not much at school (M6).*
	*I think they’re not trained. Even it is true that they treated him well, but I think they should be better trained, and the students should also be informed (M7).*

The strategies presented in Table 5 will serve as a foundation for the development of training programs and interventions led by school nurses. These may include educational sessions for staff and students; development of coping strategies for affected students; modification of harmful behaviours through inclusive school culture.

## DISCUSSION

The main findings of this study reveal the existence of various forms of bullying - physical, verbal, and through social networks - targeting transgender children and adolescents. These experiences had significant negative impacts on the participants´ biopsychosocial well-being and academic performance. Finally, this study highlights the pressing need to develop specific coping strategies to address bullying directed at transgender individuals.

This research explored the diverse experiences of bullying victimization among transgender students, along with its associated health consequences, from the perspective of both the students and their mothers. As previous studies have shown, trans students face higher rates of bullying than their cisgender peers^29^^,^^30^. Our results confirm that trans children and adolescents are subjected to multiple forms of bullying - primarily physical, verbal, and cyber bullying - in line with existing literature^14^. School-based victimization among transgender students is linked to depression, violent behaviour, school absenteeism, academic failure, school dropout, and even self-harm. These consequences can hinder their academic success and personal development^14^^,^^31^^,^^32^. Our findings also support the work of authors who emphasize the psychosocial toll that school victimization imposes on transgender youth^33^. Consequently, these students frequently experience social exclusion, which contributes to their ongoing stigmatization^19^.

Our study also identifies a lack of specific strategies to deal with school victimization among transgender youth. This reinforces the need to implement targeted initiatives aimed at protecting and fostering the development of this population in their biological, psychological and social dimensions, as suggested by other authors^20^^,^^23^^,^^34^. One such proposal mentioned by participants is the creation of a mentor figure - a trusted reference person within the school whom students can approach to share their experiences or seek help in bullying situations^23^. Similarly, the importance of including gender identity in the school curriculum is emphasized, along with implementing training sessions for students to provide appropriate information and promote understanding. These efforts aim to ensure the inclusion and social integration of transgender students, fostering a sense of belonging in the school environment^23^.

Another measure proposed is the establishment of designated spaces within schools where students can access to information and resources, overseen by a reference adult. Such a resource has been previously identified as a way to prevent school victimization and support the inclusion of transgender youth^22^^,^^23^. There is urgent need for awareness and training among the educational community, and the development of specific protocols to address bullying of trans children and adolescents^13^.

This study underscores the importance of addressing various factors that influence the experience of victimization, such as relationships with parents, teachers, and peers, as well as broader social support systems. Enhancing these areas may help transgender students develop a greater sense of social acceptance and belonging, reduce their vulnerability, and improve safety in the school environment^35^.

Other studies have noted the key role that teachers play in preventing and addressing bullying, as well as in fostering the inclusion of transgender students. Our results are consistent with these findings and further point to the need for teacher training and broader staff involvement in promoting respectful and safe educational environments1^3^^,^^35^^-^^37^.

The need to challenge rigid *gender norms* is also evident, particularly regarding access to gendered school facilities such as restrooms and changing rooms. Allowing students to use the facilities that correspond with their gender identity is essential for promoting their inclusion and supporting their gender development^22^. These findings align with previous research emphasizing the importance of dismantling heteronormative structures within schools that act as barriers to the healthy development of transgender youth. The author studied the support initiatives available in the school system for transgender students in Australia, highlighting the above-mentioned as key initiatives to ensure the proper development of their gender identity^22^.

An aspect not mentioned but present in the literature is the role of peer support groups. Some authors^38^ suggest that the presence of Lesbian, Gay, Bisexual, Transgender, and Queer (LGBTQ) student groups correlates with reduced levels of school victimization. Similary, other researches emphasize the protective and preventive functions of such groups in creating safer and more inclusive school environments^20^^,^^39^^,^^40^.

Furthermore, some of the participants raised concerns about potential stigmatization of cisgender students. In response to this, previous studies propose educational activities that cultivate values such as empathy to promote respect for gender diversity. Education in moral values - such as solidarity, empathy, humility and respect - is suggested as a tool to prevent school violence and ensure that all students enjoy equal rights and opportunities^41^.

Finally, this study emphasizes the need for greater activism and institutional action within the educational sphere to combat transphobia. It identifies strategies, supported by both transgender students and their families, aimed at preventing and addressing victimization, with the ultimate goal of creating safer and more inclusive school environments.

This study has some limitations. Sample bias: Participants were recruited through snowball sampling in Murcia and Valencia, which limits the generality of our findings to all transgender youth in Spain. Participation bias: only families willing to participate were included, potentially omitting less supported youth who may face greater risks. Lack of data triangulation: due to logistical constraints, we did not incorporate additional perspectives. COVID-19 disruptions: lockdowns interrupted in-person interviews, which were later shifted to video calls to reach data saturation.

In conclusion, transgender students in primary and secondary schools in two Spanish regions experience significant academic, psychological, and social harm due to bullying. This highlights the need for comprehensive anti-bullying measures, including policies, protocols, training, support services, and inclusive curricula designed specifically for transgender youth.

Some implications for clinical practice:

1. Education and awareness: provide students and staff with information about transgender identities to promote gender diversity understanding.
2. Supportive environment: school communities should foster a respectful, inclusive climate via mentor programs, embedded gender identity content, and activities to cultivate empathy and respect.
3. School nursing role: school nurses are well-positioned to lead interventions, deliver training, and support transgender students´ biophysical health and psychosocial needs.
4. Staff training and policy development: schools must train all personnel to recognize and intervene in transphobic bullying and establish clear, enforceable protocols to support transgender pupils´ academic and personal development.

## Data Availability

Data are available under request to corresponding author.
